# “The Safety Check”: A simple and reproducible intraoperative test to support surgeons in training during ALT flap harvesting

**DOI:** 10.4317/jced.62883

**Published:** 2025-07-01

**Authors:** Íñigo Aragón-Niño, Hailong Ma, Yue He

**Affiliations:** 1 Oral and Maxillofacial - Head & Neck Oncology Department. Shanghai Ninth People’s Hospital - Shanghai Jiao Tong University School of Medicine. Shanghai, China; 2 IdiPAZ Translational Research Group in OMFS and H&N Cancer. La Paz University Hospital. Madrid, Spain

## Abstract

The anterolateral thigh (ALT) flap is widely used in reconstructive surgery due to its versatility and favorable donor site characteristics. However, its harvest remains technically challenging, primarily due to the anatomical variability of the perforators. Experienced surgeons make intraoperative decisions based on anatomical knowledge, tactile feedback, and visual cues, but these skills may be underdeveloped in surgeons in training, increasing the risk of complications. To address this issue, we present “The Safety Check,” a simple and reproducible intraoperative test designed to assist trainees in decision-making during ALT flap harvest. This method is based on the visual assessment of arterial pulsatility along the course of perforators and the pedicle, formalizing a step that is often intuitive for experienced surgeons. The technique was applied in 52 cases over a 6-month period, with perforators monitored in three phases: before dissection, during dissection, and after dissection until flap elevation. Preliminary results showed that “The Safety Check” improved trainee confidence, facilitated the early identification of compromised perforators, and increased surgical efficiency without adding significant time to the procedure. Additionally, the method served as an educational tool, fostering active learning and intraoperative discussions. While no quantitative data were collected, qualitative feedback supported its value as an adjunct to training in perforator flap techniques.

** Key words:**Perforator flap, Microsurgery training, Intraoperative technique, Flap harvesting, Surgical education.

## Introduction

The anterolateral thigh (ALT) flap is a highly versatile reconstructive option in microsurgery, valued for its long pedicle, minimal donor site morbidity, and adaptability. However, the harvest of ALT flaps remains one of the most technically demanding procedures, primarily due to the considerable anatomical variability of the perforators (1,2). For experienced microsurgeons, decisions during flap harvest are guided by a combination of detailed anatomical knowledge, tactile feedback, and visual cues. However, these skills are not always well developed in surgeons in training or those with limited experience in perforator flap surgery. This gap in experience may result in longer operative times, increased risk of vascular compromise, and even flap failure (3,4).

To address these challenges, we introduce “The Safety Check,” a simple and reproducible intraoperative test designed to assist surgeons in training during ALT flap harvests. This method formalizes the visual assessment of arterial pulsatility, a step that experienced surgeons often perform intuitively, making it accessible and reliable for those in training. The goal is to support real-time decision-making, enhance safety, and improve the learning curve during ALT flap harvests.

## Case Report

A total of 52 ALT flap harvests were included in this protocol over a 6-month period for the reconstruction of head and neck oncological cases. All procedures were conducted by maxillofacial surgeons in the early years of their reconstructive surgery training, under the supervision of experienced microsurgeons. A total of 181 perforators were explored during these procedures and ‘’The Safety Check’’ protocol was systematically applied in each case.

The method involves monitoring perforators by visually assessing the arterial wall pulse at different points along each perforator and the pedicle. Observation can be performed with the naked eye or, preferably, using surgical loupes to improve precision.

**Figure 1:**
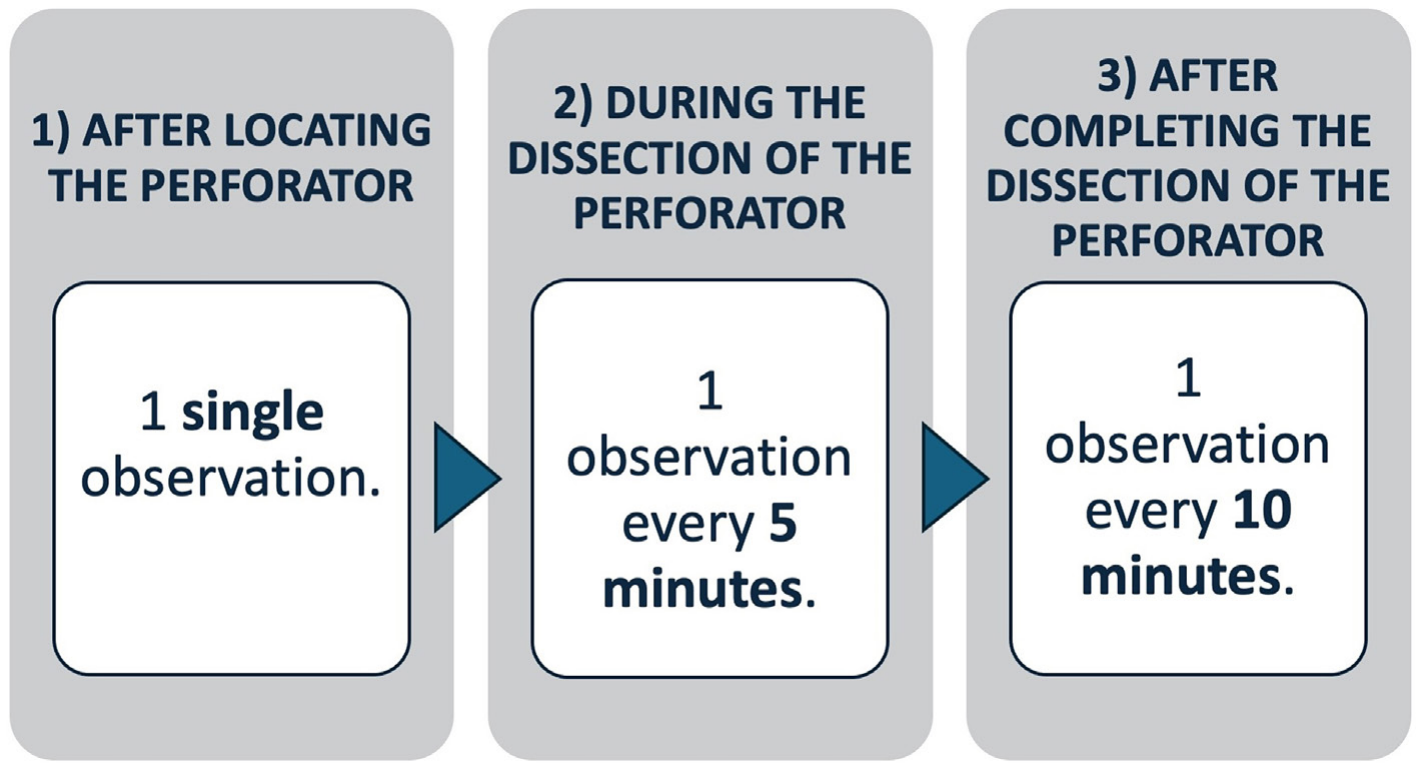
Schematic representation of the process.

The protocol consisted of three phases (Fig. 1):

Phase 1: Initial Observation: Performed after identifying perforators and before starting dissection. One observation per perforator is made to establish a baseline.

Phase 2: During Dissection: Monitoring is repeated every 5 minutes to detect early signs of perforator compromise during dissection and manipulation.

Phase 3: After Dissection Until Flap Elevation: Once the perforators are fully dissected, monitoring continues every 10 minutes until the flap is ready for pedicle division or inset.

**Figure 2:**
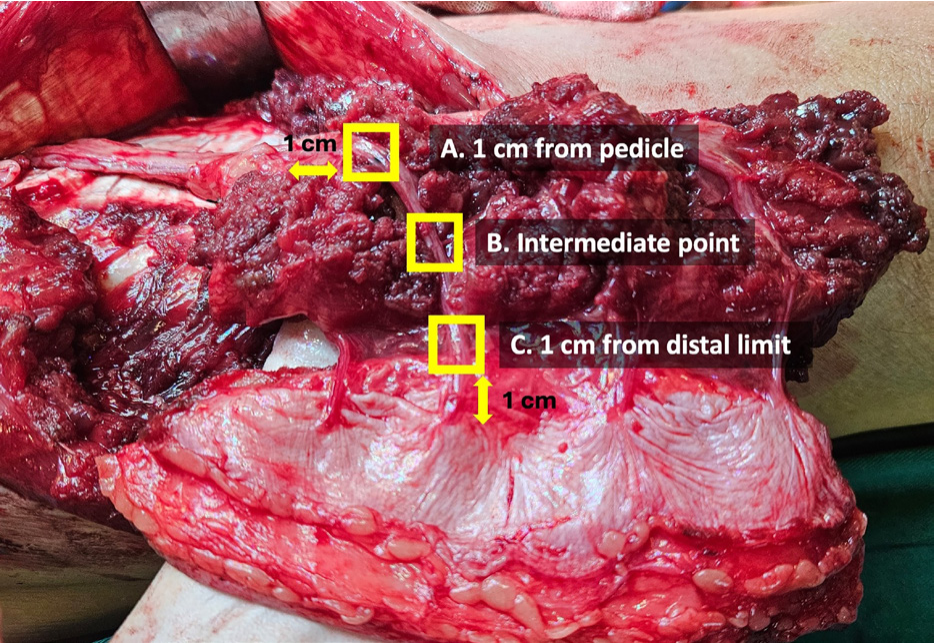
Observation reference points on the flap.

Each perforator was assessed at three anatomical locations: 1 cm from the distal end of the perforator, 1 cm from the junction with the main pedicle, and an optional intermediate point (Fig. 2). Each observation should last at least 5 seconds or until at least two consecutive pulses are detected at the same point. This assessment should be performed on all perforators of the flap, starting from the distal to the proximal perforators.

Its implementation resulted in the following observed benefits:

Confidence and Learning: Surgeons in training reported increased confidence and a better understanding of anatomical landmarks and vascular structures during the dissection process.

Identification of Compromised Perforators: The protocol facilitated the early identification of compromised perforators, especially during subtle trauma in the dissection phase, reducing the risk of unnoticed vascular compromise.

Teaching Tool: The method was successfully incorporated into the teaching process, promoting active learning and enhancing intraoperative discussions about anatomical variations and surgical techniques.

## Discussion

‘’The Safety Check’’ protocol has shown considerable promise in improving the safety and efficiency of ALT flap harvesting, particularly in a training environment. Its structured, visual assessment approach provided surgeons in training with a clear and reproducible method to monitor perforator health throughout the dissection process. This was particularly beneficial in reducing uncertainty when faced with subtle intraoperative challenges, such as poor visibility of perforators or the risk of vascular compromise.

One of the key benefits observed was the enhancement of intraoperative confidence. Surgeons in training felt more in control, as the method provided them with concrete evidence of vascular viability, which may have otherwise been difficult to assess without extensive experience. In addition, the protocol’s simplicity and minimal impact on operative time allowed it to be seamlessly integrated into the surgical workflow.

While this study did not collect quantitative data, the consistency and positive feedback from both junior and senior surgeons suggest that “The Safety Check” can be an invaluable educational tool. The feedback from senior surgeons indicated that the method promoted more productive intraoperative discussions, reinforcing the importance of real-time decision-making and anatomical knowledge.

The Safety Check is not intended to replace experienced judgment but rather to complement traditional training methods. By offering a structured, repeaTable approach to monitoring arterial pulsatility, it provides a foundation for surgeons in training to develop their intraoperative decision-making skills in a high-risk, technically demanding procedure. Additionally, the adaptability of the method makes it applicable to other perforator-based flaps, potentially broadening its utility beyond the ALT flap.

## Data Availability

The datasets used and/or analyzed during the current study are available from the corresponding author.
